# ATria: a novel centrality algorithm applied to biological networks

**DOI:** 10.1186/s12859-017-1659-z

**Published:** 2017-06-07

**Authors:** Trevor Cickovski, Eli Peake, Vanessa Aguiar-Pulido, Giri Narasimhan

**Affiliations:** 1 Bioinformatics Research Group (BioRG) & Biomolecular Sciences Institute, School of Computing & Information Sciences, Florida International University, 11200 SW 8th St, Miami, 33196 FL USA; 2 Department of Computer Science, Eckerd College, 4200 54th Avenue South, Saint Petersburg, 33711 FL USA

**Keywords:** Centrality, Biological network, Microbial social network, Economic payoff

## Abstract

**Background:**

The notion of *centrality* is used to identify “important” nodes in social networks. Importance of nodes is not well-defined, and many different notions exist in the literature. The challenge of defining centrality in meaningful ways when network edges can be positively or negatively weighted has not been adequately addressed in the literature. Existing centrality algorithms also have a second shortcoming, i.e., the list of the most central nodes are often clustered in a specific region of the network and are not well represented across the network.

**Methods:**

We address both by proposing *Ablatio Triadum* (ATria), an iterative centrality algorithm that uses the concept of “payoffs” from economic theory.

**Results:**

We compare our algorithm with other known centrality algorithms and demonstrate how ATria overcomes several of their shortcomings. We demonstrate the applicability of our algorithm to synthetic networks as well as biological networks including bacterial co-occurrence networks, sometimes referred to as *microbial social networks*.

**Conclusions:**

We show evidence that ATria identifies three different kinds of “important” nodes in microbial social networks with different potential roles in the community.

## Background

The concept of *centrality* is foundational in social network theory and its underlying motivation is to find the most important or “critical” nodes in a large complex social network [1]. In this type of network, one may be interested in finding the most influential or the most popular individual. A search engine may want to rank the hits resulting from a search, depending on how well linked it is in the network. In a terror network, an agency may be interested in finding the ringleader or the top leadership. Thus, “centrality” can have multiple meanings, and different metrics and methods are worth exploring.

With the advent of systems biology approaches, large-scale biological networks have become commonplace. Gene regulatory networks [2] model the interactions between genes, while protein-protein interaction (PPI) networks [3] represent the interaction of proteins. *Microbial* social networks [4–6] attempt to model the complex interactions between microbes within a microbial community, such as those that inhabit the human gut or those that can be found in diseased coral.

**Fig. 1:**
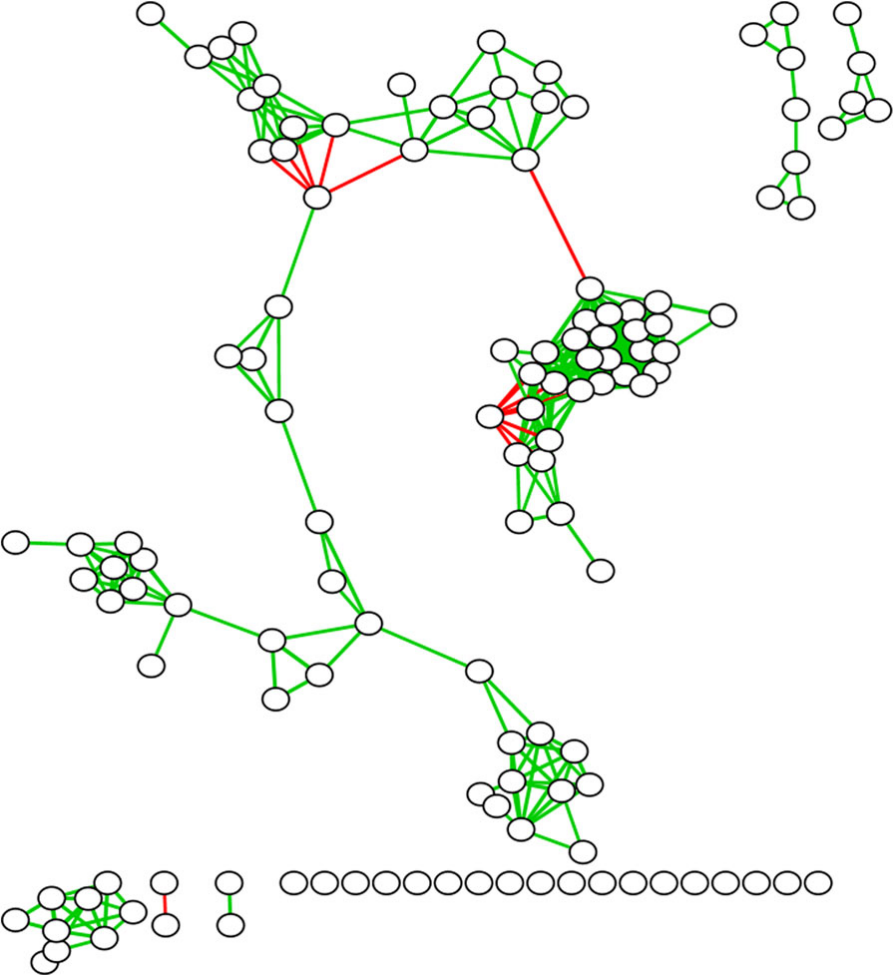
Bacterial Co-Occurence Network. An example of a bacterial co-occurrence network obtained from a lung microbiome study. Nodes represent bacterial taxa. Green (resp. red) edges represent positively (resp. negatively) correlated co-occurrence patterns

It is well known that microbes in a community interact. These interactions may occur through the use of quorum sensing molecules, other signalling molecules, metabolites and/or toxins [7–9]. However, lacking the access to precise interaction information in sampled microbial communities, it has been suggested that bacterial co-occurrence networks inferred from metagenomic studies are a crude form of microbial social networks [4, 6]. A bacterial co-occurrence network [10] is an undirected, weighted network with nodes that represent bacterial taxa present in the community and edges that correspond to how strongly the two taxa tend to co-occur (i.e., co-infect) in the sampled communities. Edge weights can be positive or negative lying in the range [−1,+1]. We show an example of this in Fig. 1, using data from a lung microbiome study. Green edges indicate positive correlations and red edges indicate negative ones, with edge thickness indicating strength of correlations. We visualize results using the Fruchterman-Reingold algorithm [11] within Cytoscape [12]. Even a cursory visual inspection of the network suggests the presence of dense subgraphs representing strongly co-occurring groups of bacteria (referred to as *clubs* [6]). In co-occurence networks, strong green edges suggest the likelihood of *cooperation*, while strong red edges suggest *competition*.

The following questions arise naturally in these investigations. Is it possible to identify bacterial taxa that drive or control the behavior of the community through their interactions? Can the first infectors or colonizers of the community be identified? What is the effect of disrupting a node or edge of such a biological network? All the above questions highlight the importance of studying central nodes in biological networks [13]. We suggest three notions of centrality that are potentially important to biological networks, and especially to microbial social networks. The work in this paper addresses all three notions:

1. For each club (high density subgraph), we refer to a dominant node as a *leader* node [14], or an entity responsible for connecting many individuals and driving the behavior of the club.
2. We define a *villain* node as one that has many strong negative edges to a club. Unity against a common enemy is a frequent theme in social networks [15].
3. Nodes that connect two or more dense subgraphs (clubs) are referred to as *bridge* nodes. In general social networks, this would correspond to someone who has the ability to link different social circles [15].

Centrality concepts [16, 17] can be classified into three categories: degree centrality, closeness centrality, and betweenness centrality. *Degree* centrality assumes that the most important nodes have high connectivity or degree. It is useful in identifying popular individuals in a social network. *Closeness* centrality interprets centrality with respect to a distance metric, identifying nodes that are centrally located. This would be useful in identifying where to place an important network resource (e.g., fire station or database server). *Betweenness* centrality defines a central node as one that lies on many shortest paths. Betweenness centrality would help identify important junctions in a complex train or information flow network. Other approaches define an entity’s centrality by the importance of its friends in the social network. Eigenvector-based approaches [16] for centrality extend the ideas of degree and closeness centrality by explicitly defining the centrality of a node in terms of the importance of its neighbors. Google’s *PageRank* algorithm [18] is an example of this approach. In this paper, we will propose an algorithm that combines and generalizes these concepts.

Most of these approaches also generalize to *weighted* social networks, where edge weights represent the strength of the relationship or influence between nodes. Distance-based methods like closeness and betweenness extend trivially. Degree can be generalized to weighted degree. The original version of PageRank assumes edge weights of 0 and 1, but subsequent attempts have been made to generalize the algorithm to weighted networks [19]. However, not many generalize readily to networks with *negative* edge weights, which is an important characteristic of real social networks because it helps distinguish between “indifference” and “dislike”. *PageTrust* [20] extends PageRank to handle negative edges but, since all final centralities are positive, it becomes difficult to distinguish a villain vs. a node with few friends as they both have low values. The PN-Centrality algorithm [21] of Everett and Borgatti fixes this problem but, as an eigenvector-based approach, tends to be biased toward nodes in highly dense subgraphs, thus distorting centrality information. Degree centrality has this same difficulty with cliques or dense subgraphs having many strong edges. Closeness centrality tends to have a cluster of nodes with high centrality with values decreasing from there, biasing a particular area of the network. Betweenness centrality is better at identifying bridges but not leaders or villains.

In this work we present *ATria*, an iterative centrality algorithm that addresses the shortcomings mentioned above and combines aspects of economic theory, social network theory, and path-based algorithms [22]. We investigate methods that avoid the above shortcomings by iteratively removing nodes with highest centrality along with some of the neighborhood edges before finding the node with the next highest centrality, using social network theory to determine the appropriate edges to remove. The goal of ATria is to find leaders, villains and bridges within a signed, weighted social network. We will verify that ATria is able to produce these results by testing a wide-range of networks including some simple synthetic examples, a scale-free network [23], and biological networks, such as gene expression, PPI, and microbial social networks.

## Methods

Our proposed algorithm incorporates economic theory to reflect the fact that our interest in leader, villain and bridge nodes is based on their benefit (good or bad) to the network as a whole. Conjecturing possible interpretations, a leader node can be interpreted as a dominant member of a club, by being a major producer or consumer of some resource (e.g., a metabolite) that benefits other club members. A villain node may either represent a common enemy against which members of a club unite, or the producer of some byproduct (e.g., toxin) that is harmful to all members of a club. Bridge nodes may represent taxa that provide a beneficial (or harmful) resource to more than one club. Alternatively, they could be an important part of a cascade of events in a process.

Our starting point for an economic model is the *Payoff Model* proposed by Jackson and Wolinsky [24], which analyzes the efficiency and stability of an economic network where every node in the network provides some *payoff* to every other node. They use this approach to determine nodes that receive the highest *pay* (meaning, the largest benefit from their connections), representing payoff for a node *i* in network *G* with uniform edge weights 0<*δ*<1 by the following:

1 $$ u_{i}(G) = w_{ii} + \sum\limits_{j \ne i} \delta^{t_{ij}} w_{ij} - \sum\limits_{j: ij\in G} c_{ij}   $$

In the above model, *w*_*ii*_ represents an amount of starting “capital” for node *i*. They use *w*_*ij*_ to represent an innate significance of node *j* to node *i*. The second term multiplies *w*_*ij*_ by a factor that is exponential in *t*_*ij*_, the number of links in the shortest path between *i* and *j*. If 0<*δ*<1, this term ensures that the payoff contribution for node *i* is higher for nodes *j* that are closer. The shortest path between *i* and *j* will thus result in the highest pay for *i* from *j*, and is the only pay that is used. The final term *c*_*ij*_ represents a cost (instead of a payoff) for node *i* to maintain a direct connection to a neighboring node *j*. In summary, closer nodes contribute more, but direct connections incur a cost.

The intuition behind the connection between the payoff model and centrality is as follows. If (a) all nodes start with the same capital (i.e., *w*_*ii*_=0), (b) nodes do not contain any intrinsic value to one another before the algorithm runs (i.e., *w*_*ij*_=*w*_*ji*_=1), and (c) there is no cost to maintain direct connections (i.e., *c*_*ij*_=0) then the network is *symmetric*. This implies that in an undirected network the amount of “pay” received by a node (positive or negative) is the same as the amount they are providing to other nodes. Pay thus becomes a direct measurement of a node’s benefit to the network.

### Extended payoff model

In designing our algorithm ATria, we take the symmetric algorithm by Jackson and Wolinsky and extend it in the following ways to encapsulate more general social networks:

1. We allow for edge weights to be non-uniform. Therefore, instead of all weights being equal to *δ*, the edge weights are 0<*δ*_*ij*_<1. As a consequence, in the second term of Eq. 1 we replace $\phantom {\dot {i}\!}\delta ^{t_{ij}}$ by the product of the *δ* values along the path of maximum pay between node *i* and node *j*.
2. We incorporate negative edge weights, under the limited assumption that all weights are in the range −1<*δ*_*ij*_<1. With negative edges, a node receives a *negative* benefit from its connection with a neighbor. However, a path with two negative edges will result in a positive payoff, since the total payoff from a path is the product (not sum) of its edge weights.
3. Centrality is computed iteratively. The most central node is found first, with ties broken arbitrarily. This node is then deleted along with some of the edges in its neighborhood. The centrality values are then recomputed for all the nodes. Although ties are broken arbitrarily, this does guarantee that the list of the most central nodes are not occupied by nodes that are all close to each other. Hence, ATria will find central nodes from all across the network.

Our modified equation, after removing *c*_*ij*_, is thus:

2 $$  u_{i}(G) = \sum\limits_{j \ne i} P(i,j),  $$

where *P*(*i,j*) is the path of maximum pay magnitude between *i* and *j*.

A major deviation from the payoff model is that our algorithm computes the centrality values incrementally as opposed to all at once. Therefore, even if the node with the highest *u*_*i*_(*G*) value may be judged the most central node in the first iteration, the node with the second highest value in the first iteration will not end up as the second most central node, unless it is the highest in the second iteration.

**Fig. 2:**
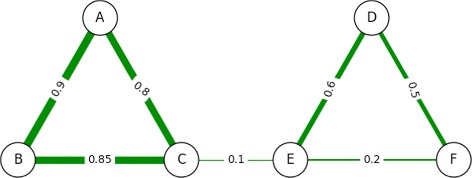
Two-Triad Social Network. A sample social network with two strongly connected triads {*A,B,C*} and {*D,E,F*}

Consider the example in Fig. 2. In this network, the payoff model would compute node *B* as being the most central to the network, but then would compute *A* as the second most central and *C* as the third most central. While this may make sense for the payoff model itself (both *A* and *C* receive large benefits from *B*), it has some shortcomings from the point of view of centrality to say that *A* and *C* are the next most important nodes, since most of their pay comes as a result of *B*. ATria would first find *B* as the most central node as a *leader* of the first triad, but it would then find *D* as the second most central node as a *leader* of the second triad.

This happens because the edges incident on *B* are deleted after *B* is determined as having the highest centrality. The logic here is to remove all dependencies on the most central node before computing the next most central node. Also for every triad involving two of these incident edges, we remove the third edge if both incident edges have the same sign and the third edge is positive. This is backed up by social network literature [15], which states that two nodes with a mutual friend (in this case the leader *B*) or enemy (a villain) will tend to become friends as a result, meaning their connection is *coincidental* and resulting not from their own importance but the importance of the leader or villain. Such a triad with an even number (zero or two) of negative edges is said to be *stable*, a necessary condition for social network balance.

#### Incorporating non-uniform edge weights

**Fig. 3:**
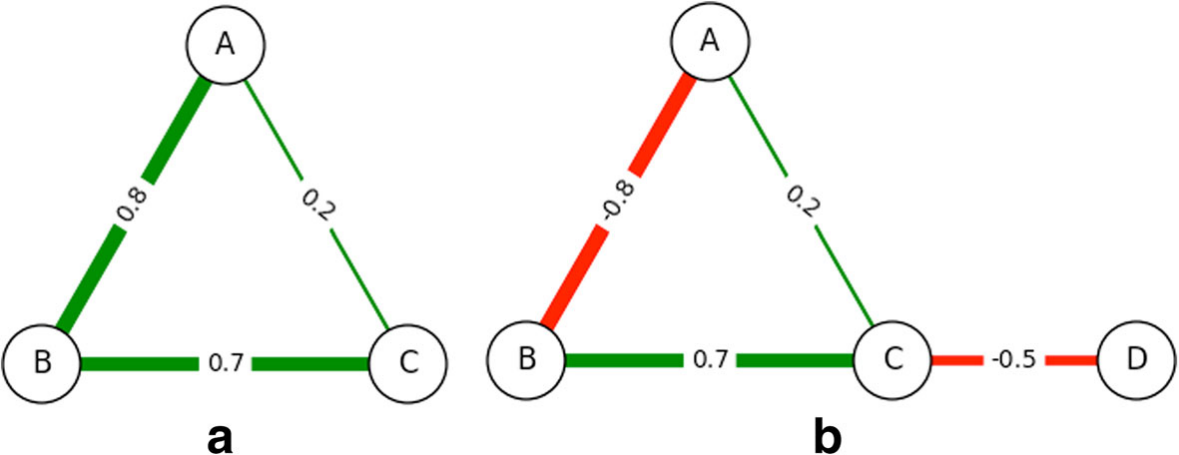
Non-Uniform Weighted Networks. **a** An example social network with non-uniform positive edge weights. In this situation, the payoff between *A* and *C* is larger via their indirect connection through *B* (0.56) compared with their direct connection to each other (0.2). **b** An example network with non-uniform positive and negative edge weights. Nodes can now gain and lose from each other

The first change that we make to the Payoff Model, as mentioned, is incorporating non-uniform edge weights. In the unweighted (or uniformly weighted) case, the shortest path between *i* and *j* is guaranteed to have the fewest number of edges; this may not be true any longer, as illustrated in Fig. 3(a).

To incorporate this change, we use a modified form of Dijkstra’s Algorithm. In particular, the length of a path is the product of its lengths, and the best path is the one with the maximum (not minimum) product. Note that since all edge weights are between 0 and 1, the products can only decrease in magnitude as the path gets longer. Such a modified Dijkstra’s algorithm when started at node *i*, will help compute *P*(*i,j*) for all *j*, thus computing *u*_*i*_(*G*) (see Eq. 2).

#### Incorporating negative edge weights

When negative edge weights are present in the network, we have a possibility for nodes to gain and lose from each other depending on the path along which the effect takes place. Similar to the path of maximum gain, we consider the path of maximum loss as more significant to a node’s centrality as opposed to one of a smaller loss. However, there may be pairs of nodes between which there is a positive length path as well as a negative length path. Consider the network in Fig. 3(b). There are two paths between *A* and *D*: *A* – *C* – *D*, and *A* – *B* – *C* – *D* with path lengths of 0.2×−0.5=−0.1 and −0.8×0.7×−0.5=0.28, respectively. One causes a gain, the other incurs a loss.

Dijkstra’s algorithm is modified so that for every starting node *i*, we simultaneously keep track of two quantities: the length of the path of highest gain to node *j*, and length of the path of highest loss to node *j*. This covers situations like in Fig. 3(b) where the path of highest gain from *A* to *D* includes a path of highest loss from *A* to *C* and a path of highest loss from *C* to *D*. We then modify the RELAX step in Dijkstra’s algorithm [25] as follows: when relaxing edge (*j,k*), if its weight is positive, then we use the maximum gain due to node *j* to update the maximum gain due to node *k* and the maximum loss due to node *j* to update the maximum loss due to node *k*. On the other hand, if its weight is negative, then we use the maximum gain due to node *j* to update the maximum loss due to node *k* and the maximum loss due to node *j* to update the maximum gain due to node *k*.

To incorporate both gain and loss, we modify our payment equation to set *P*(*i,j*)=*G*(*i,j*)+*L*(*i,j*), where *G*(*i,j*) is the length of the path of maximum gain between *i* and *j* and *L*(*i,j*) is the length of the path of maximum loss (negative or zero). So our final payment equation for ATria becomes:

3 $$  u_{i}(g) = | \sum\limits_{j \ne i} G(i, j) + L(i, j) |  $$

## Results and discussion

In order to test ATria, we run our algorithm on sample networks alongside five other centrality algorithms: betweenness, closeness, degree, and the eigenvector-based approaches PageRank (PageTrust if the graph has negative weights) and PN. To be fair we use weighted degree centrality, and for running Dijkstra’s algorithm for closeness and betweenness centrality we compute distance by taking the negative logarithm of the absolute value of an edge (so larger edge magnitudes carry smaller weights, yielding shorter paths).

### Networks with cliques

#### Single clique

**Fig. 4:**
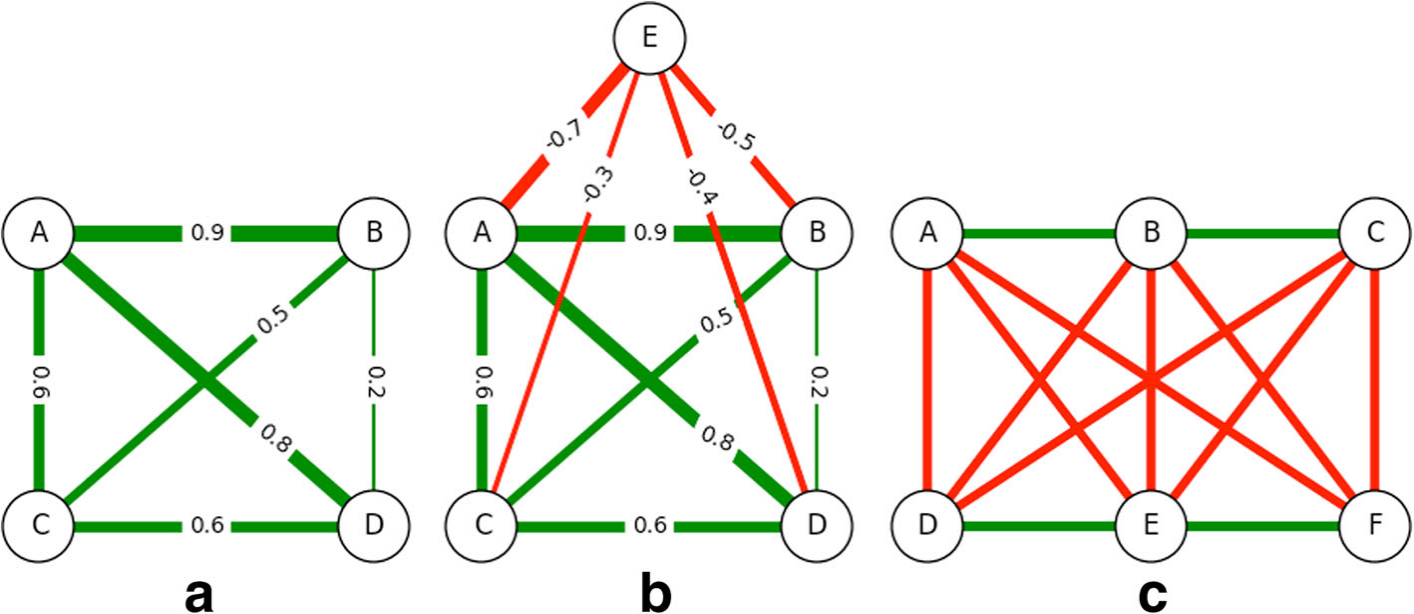
Weighted Cliques. **a** A weighted four-clique with leader *A*, **b** Clique **a** with a villain *E*, **c** A clique of rival groups. The same node can be a leader and a villain

We begin by studying weighted cliques. The first is a non-uniform weighted clique of size four with a leader *A* (in Fig. 4(a)). The second is the same clique but with the addition of a villain node *E* (Fig. 4(b)). Finally, we show a uniform-weighted clique of rival groups in Fig. 4(c), where the most central node will be a leader to one group and a villain to the other. While ATria agreed with all other algorithms on the most central node for all three examples, only ATria clearly identified *A* as the leader in (a), *E* as the villain in (b), and *A* (arbitrarily, but the point remains) as leader and villain in (c). It does this by setting all other centralities to zero, thus assuming that all remaining connections result from connections to these nodes.

#### Multiple cliques

**Fig. 5:**
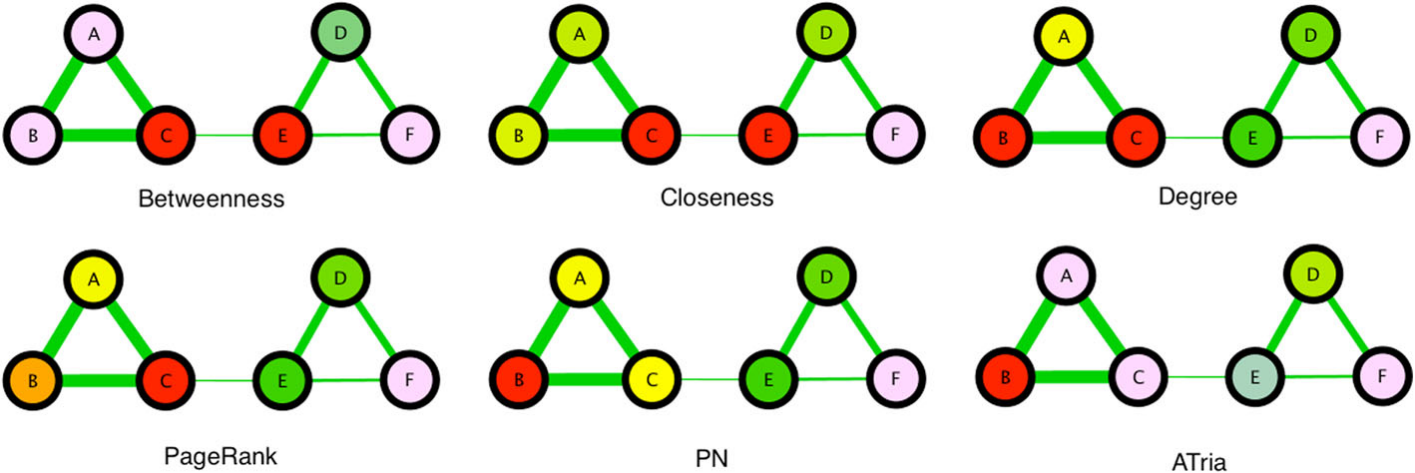
Comparison on Two-Triad Social Network. A comparison of ATria with five other centrality algorithms on the network from Fig. 2. Red nodes are the most central

Figure 5 shows our first example of a multiple-clique network, which is the non-uniform weighted network from Fig. 2 that has two positive triads connected by a weaker positive edge. In this figure we compare the results of all six algorithms, color coding individual centrality values against a normal distribution (red=maximum, violet=minimum, blue and green respectively two and one standard deviations left of the mean, yellow one to the right, orange two to the right). Degree, PageRank and PN all biased the tighter-connected first triad, while betweenness and closeness biased the triad bridges. As discussed earlier, ATria computed *B* as most central (first triad leader), and *D* as second (second triad leader). *E* is then arbitrarily chosen as third over *C*. ATria thus favors leaders above bridges if triad edges are stronger than their connections. This holds independent of the sign of the connections. If the connection edge *CE* was stronger than the triads, ATria would choose *C* as most central for a positive *CE* (*C* is in the tighter triad and has closer friends) and *E* as most central for a negative *CE* (for this same reason, more nodes are harmed by its competition with *C*).

**Fig. 6:**
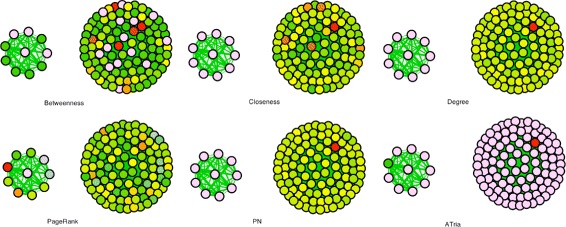
Comparison on Two Varying-Sized Cliques. Results when running ATria and the other centrality algorithms on two cliques, one of size 10 and the other of size 100

Figure 6 shows a more extreme example, which contains one clique of ten nodes and another of one hundred nodes. All edges have random positive weights in the range (0,1). Note that ATria is able to immediately pick out both leaders, ranking the leader of the larger clique with a much higher centrality than that of the smaller. All other approaches tend to favor one of the two cliques. We summarize these results in Table 1.

**Table 1:** Top two central nodes found by ATria and other centrality algorithms on simple networks (*=leader, +=villain). If only one node is listed, all others have centrality zero. Braces indicate a tie. For the weighted 4-clique we ran one example with a leader node and one with a villain. For the two cliques, *N(i)* indicates some neighbor of node *i*, which may vary with the algorithm

	Betweenness	Closeness	Degree	PageRank	PN	ATria
Wt 4-Clique 1	A*	A*, D	A*, C	A*, C	A*, C	A*
Wt 4-Clique 2	*E*+	*A, D*	*E+, A*	*A, C*	*A, C*	*E+*
Rival Groups	*{A, E}*, *{B, F}*	all nodes	all nodes	all nodes	all nodes	*A*
Two Triads	*{C, E}*, D*	*{C, E}*, B*	{B*, C}, *A*	*C*, {A, B*}	B*, *{A, C}*	B*, D*
Two Cliques	A*, N(A)	A*, N(A)	A*, N(A)	A*, N(A)	A*, N(A)	A*, B*

### Synthetic network with clubs

**Fig. 7:**
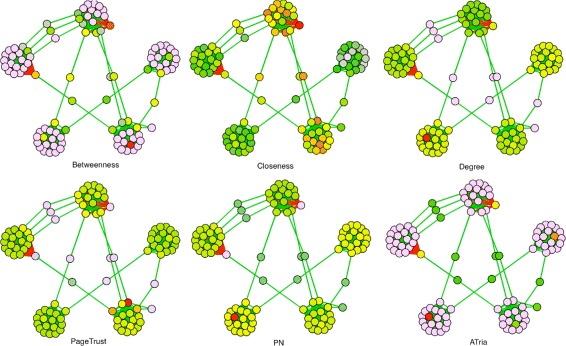
Comparison on Synthetic Network. A comparison of ATria with five other centrality algorithms on a synthetic network with five cliques (three with a leader, two with a villain), plus some bridge nodes

We now develop a synthetic network to illustrate the type of network for which ATria is most beneficial, with five cliques of random sizes between 16 and 20. We randomly choose one leader node for each of three of the cliques, and one villain node for each of the other two. We connect leaders to their clique using random edge weights in the range [0.85,1), and villains using (−1,−0.85]. Edges between other nodes are between 0.75 and the lower of the two edges with the leader or villain. We choose a number of bridge nodes equal to half the size of the largest clique and connect them to a random node in two random cliques using a random weight in the range [0.75,1). We run all six algorithms on this network and show our results in Fig. 7. As can be seen, ATria was able to immediately pick out leaders, villains and bridges and set all other centralities to zero.

This situation also illustrates challenges with other centrality approaches for this type of network. Betweenness was the only other algorithm able to somewhat separate leaders, villains, and bridges since in this example they reside on most high pay paths, but for this same reason also counted clique nodes connected to bridges (in some cases even above leaders and villains). Closeness centrality biased the cliques connected by the most bridges, and degree biased the tightest connected cliques. PageTrust and PN found the two villains (low centralities by design) and PN also found the top two leaders (the second less obvious), but then biased their cliques and lost the third. We summarize these results in Table 2.

**Table 2:** Comparison of ATria’s results with those other algorithms on a 102-node synthetic network with five cliques, three with leaders *A, B, C*, two with villains *D, E* and bridge nodes *F-O* connecting cliques

Node	Betweenness	Closeness	Degree	PageTrust	PN	ATria
A (Leader)			2		2	2
B (Leader)	9		1		1	1
C (Leader)	1	3		4		5
D (Villain)	3		3	88	102	4
E (Villain)	2	1		102	101	3
F (Bridge)			101	98	98	6
G (Bridge)			96	95	94	10
H (Bridge)			100	101	97	13
I (Bridge)			95	94	93	9
J (Bridge)	15		98	91	96	15
K (Bridge)	8		97	99	97	11
L (Bridge)			99	97	96	12
M (Bridge)	5		93	96	91	7
N (Bridge)		99	102	100	99	14
O (Bridge)	13	8	94	93	100	8

Final rankings of any nodes *A-O* found in the top or bottom 15

### Biological networks

**Fig. 8:**
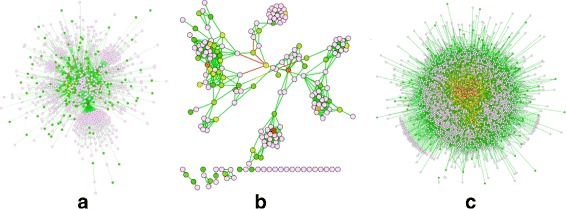
Comparison on Biological Networks. Results of ATria on **a** a 1,000-node scale-free network, **b** a gene co-expression network from a species of oyster, and **c** a yeast PPI network

We now demonstrate ATria’s results on three types of biological networks. The first, shown in Fig. 8(a) is a synthetic scale-free network of 1000 nodes. We use this as an overarching example of a network that is common across many areas of biology, including PPIs, cell signalling pathways [26], and neural networks [27]. The second, in Fig. 8(b), is a gene co-expression network (GEO:GSE31012) from a species of oyster under different salinity conditions. Finally as our largest example in Fig. 8(c), we run a yeast PPI [28] (BioGrid:S288c) consisting of 5526 nodes. Note that the PPI is by definition uniformly weighted and positive, since proteins either interact or do not interact.

Scale-free networks are known for the presence of critical *hub* nodes, which ATria also ranks with the highest centrality. The co-expression network shows that with more realistic biological data, ATria can still find leaders and villains across the network. The transcription factor *Nuclear Y-Subunit Alpha* (NYFA, [29]) was ranked #7 by ATria. This was found first by degree and PN centrality, but no other algorithms found transcription factors in their top ten. However, while degree and PN centrality then biased central nodes around this transcription factor, ATria was able to find a protein TRIM2 (#2) from the Tripartite Motif (TRIM, [30]) family, which no other algorithm found. TRIM2 helps bind the molecule *Ubiquitin* to proteins as a tag for later modification [31]. ATria discovered Ubiquitin itself as #4 in the yeast PPI. A specific type of modification for which Ubiquitin binds to proteins is degradation in the proteasome, and ATria also found Rpn11 (#7), which is responsible for removing Ubiquitin from proteins before entering the proteasome [32]. These results exhibit agreement with Cicehanover, Hershko and Rose in their discovery of Ubiquitin-mediated proteolysis and its regulation of numerous critical cellular processes including the cell cycle [33], helping them win the 2004 Nobel Prize in Chemistry.

### Microbial social network

**Fig. 9:**
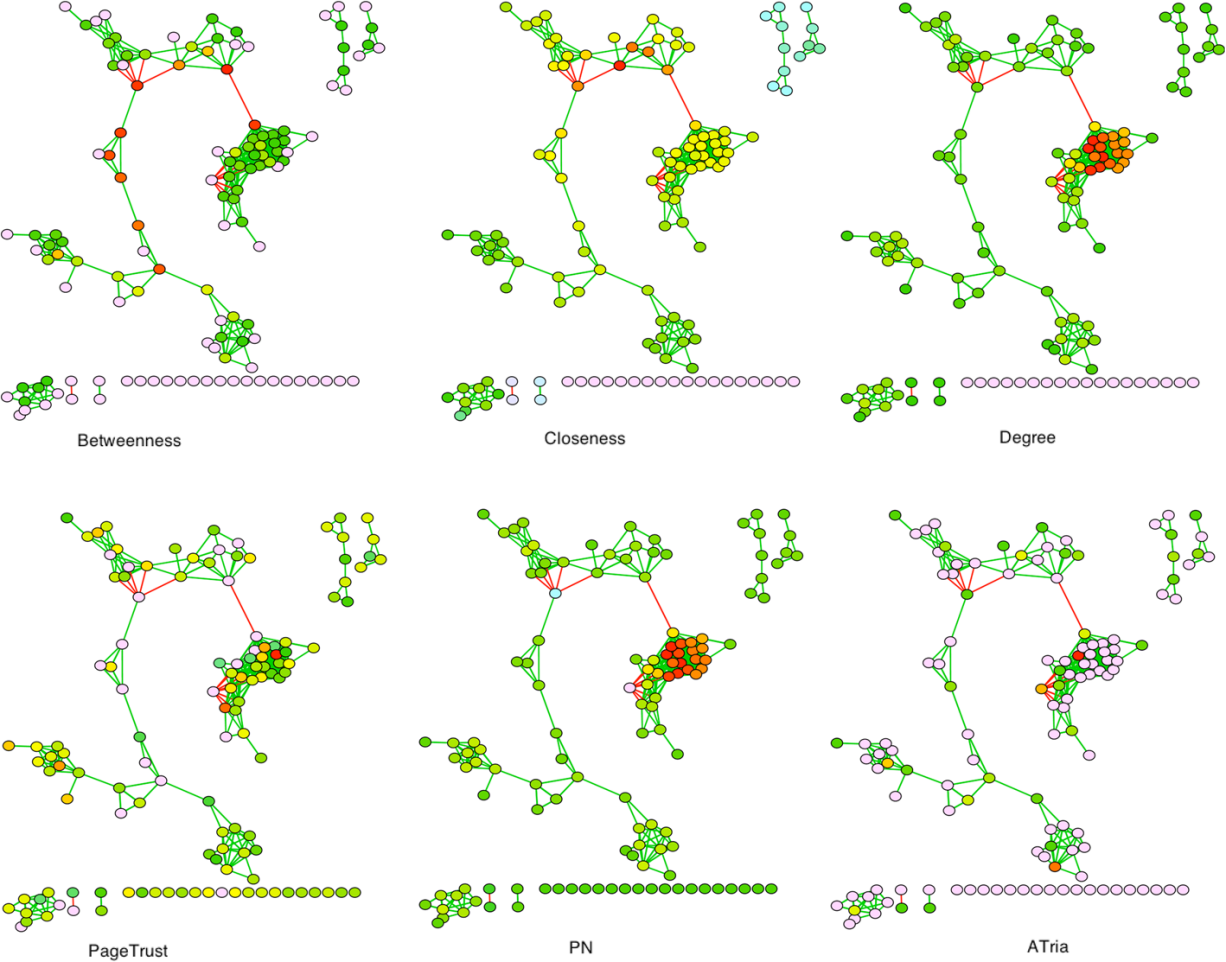
Comparison on Microbial Social Network. A comparison of ATria to the other five centrality algorithms on the co-occurence network assembled from lung microbiome data, from Fig. 1

We now show the results of ATria and the five other centrality algorithms on the co-occurence network assembled from human lung microbiome data, from Fig. 1. These results are shown in Fig. 9.

For this network, both degree and PN centrality restricted the highest ranked nodes to the tightest club in the center of the network. Closeness centrality tended to bias the center of the largest connected component, with centrality decreasing as nodes were more out of this loop. Betweenness centrality was heavily biased towards bridges in the largest connected component. The only other algorithm that was able to find central nodes in multiple clubs was PageTrust; however, ATria was able to better isolate one or two nodes in each club, followed by the bridges.

Based on the results of ATria, the bacterial taxa most likely to be producing a critical metabolite would be: *F. Burkholderiaceae* (the most central node, leader of the tightest club in the middle), *F. Erysipelotrichaceae* (#2, leader of the club just to the south), *Bifidobacterium* (#4, leader of the club to the southwest), and *Atopobium* (#6, leader of the southernmost component). *F. Prevotellaceae* (#3) is a villain of the tightest knit club which is likely to be in competition for a resource (possibly the same metabolite) that many bacteria in this club need. Bridge nodes such as *Prevotella* (#5, connecting many nodes in the two northernmost clubs) and *Selenomonas* (#8, part of a central bridge connecting the southwestern clubs to the largest connected component) could be producing a metabolite that benefits multiple clubs. Interestingly, ATria also found *C.Gammaproteobacteria* (#7), which is an enemy bridge between the largest club and the rest of this largest connected component. This could indicate competition with its counterpart *Fusobacteria* as critical to the network structure.

## Conclusions

Our results demonstrate that the application of economic models using payoffs can be useful to computing *centrality* in a signed and weighted social network when finding important leader, villain and bridge nodes. We built ATria as an iterative extension of a payoff model using social networking principles and in the process overcome shortcomings of existing algorithms for computing centrality, identifying central nodes across the network as opposed to many in the same vicinity. We verifed these results using scale-free networks and synthetic networks with both positive and negative edge weights, both of which are particularly relevant in biological networks, and finally real biological networks including a bacterial co-occurence network (or Microbial Social Network).

As future work, we would like to explore extensions of ATria to directed networks, as while uncommon in the social networking field would be useful when applied to biological networks. We also would immediately like to explore the idea of interference [34] to show and analyze the effects of removing ATria’s highly central nodes from our networks. Finally, since the time complexity of ATria is more expensive than other centrality algorithms (see Table 3) due to recomputing centralities *n* times in the worst case, we have developed a module of ATria for the Graphics Processing Unit (GPU) and plan on releasing this open-source as part of a larger microbial analysis pipeline.

**Table 3:** Time complexity of ATria, compared to other centrality algorithms

Algorithm	Time complexity
Betweenness	*O*(*n* ^3^)
Closeness	*O*(*n* ^3^)
Degree	*O*(*n* ^3^)
PageTrust	*O*(*i*·*n* ^3^)
PN	*O*(*i*·*n* ^3^)
ATria	*O*(*n* ^4^)

For eigenvector-based algorithms, *i* is the number of iterations that it takes to converge

## Abbreviations

ATria: Ablatio Triadum
GPU: Graphics processing unit
PPI: Protein-Protein Interaction
TRIM: Tripartite motif
